# Hyaluronic acid in viscous malignant mesothelioma pleural effusion

**DOI:** 10.1002/rcr2.694

**Published:** 2020-12-03

**Authors:** Hui Min Cheah, Deirdre Fitzgerald, Amber Louw, Jenette Creaney, Y. C. Gary Lee

**Affiliations:** ^1^ Pleural Medicine Unit Center for Respiratory Health, University of Western Australia Perth WA Australia; ^2^ Medical School University of Western Australia Perth WA Australia; ^3^ Department of Respiratory Medicine Sir Charles Gairdner Hospital Perth WA Australia; ^4^ PathWest Laboratory Medicine QEII Medical Centre Perth WA Australia; ^5^ School of Medical and Health Sciences Edith Cowan University Joondalup WA Australia; ^6^ National Centre for Asbestos Related Diseases Perth WA Australia

**Keywords:** Hyaluronic acid, hyaluronidase, malignant pleural effusion, mesothelioma, viscosity

## Abstract

Malignant pleural effusion (MPE) is common with mesothelioma. We report two cases of extraordinarily viscous MPEs associated with mesothelioma. The viscosity prohibited spontaneous gravity‐dependent drainage via indwelling pleural catheters. Our ex vivo experiments found very high hyaluronic acid (HA) content within the fluid. Treatment of the fluid with hyaluronidase, but not with deoxyribonucleases, significantly reduced fluid viscosity. The results provide proof that HA can contribute to high viscosity of pleural fluid in mesothelioma. Research into strategies of counteracting HA properties in the management of MPEs may provide further insight.

## Graphical abstract

We report two cases of extraordinarily viscous malignant pleural effusion (MPE) in mesothelioma patients. Our ex vivo experiments attributed the viscosity to the high hyaluronic acid (HA) content within the fluid, which could be significantly reduced with hyaluronidase.

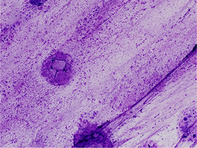

## Introduction

Pleural effusions affect over 90% of patients with malignant pleural mesothelioma. The malignant pleural effusion (MPE) commonly appears serosanguinous or haemorrhagic, and can be evacuated using a range of devices, such as aspiration needles, chest tube, or indwelling pleural catheter (IPC). We report two cases of extraordinarily viscous MPEs in mesothelioma patients. Our ex vivo experiments attributed the viscosity to the high hyaluronic acid (HA) content within the fluid, which could be significantly reduced with hyaluronidase.

## Case Report

### Patient 1

A 79‐year‐old man presented with a left‐sided pleural effusion which was noted as viscous on the initial therapeutic aspiration. Cytology confirmed malignant mesothelioma. Upon recurrence of the MPE, an IPC (Rocket, UK) was inserted but the thick fluid prohibited spontaneous drainage. He was referred to our centre for further management. The fluid was partially evacuated by connecting the IPC via a connector to a 60‐mL syringe through which manual suction could be applied. The viscosity of the fluid prohibited usual laboratory biochemical analyses of the fluid. In an attempt to reduce fluid viscosity to clear the remaining fluid, two doses of 5 mg of deoxyribonuclease (DNase) was instilled over two days via the IPC, after which ~300 and ~200 mL of fluid were drained. The underlying lung re‐expanded on chest X‐ray (CXR) and talc slurry pleurodesis was administered via the IPC.

**Figure 1:**
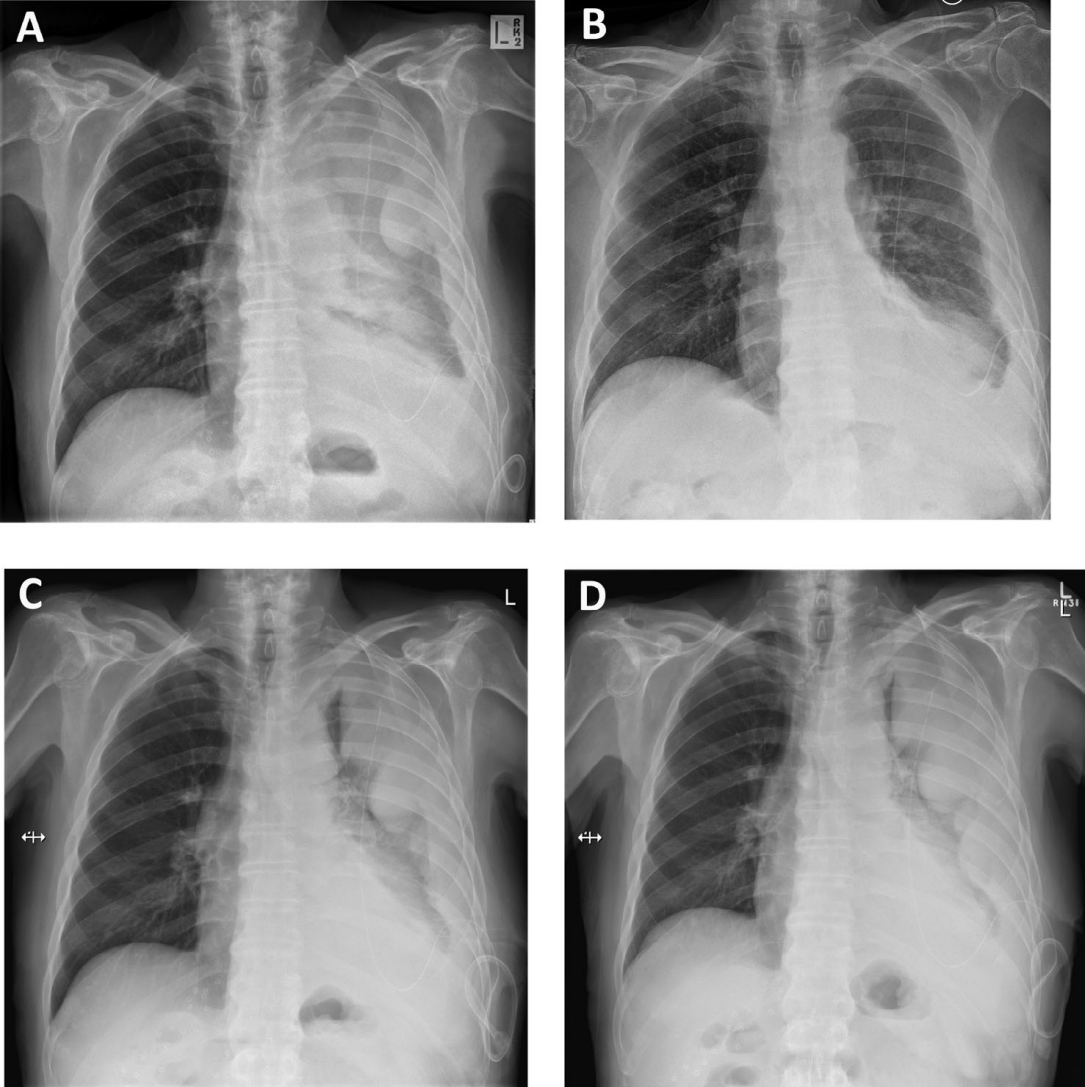
The viscous fluid of Patient 1 made drainage via indwelling pleural catheter (IPC) difficult, resulting in large loculated collections of pleural fluid, requiring hospital admission. The chest radiograph (A) showed almost complete opacification of the left hemi‐thorax which cleared with intrapleural tissue plasminogen activator (tPA) and deoxyribonuclease (DNase) therapy (B). However, the benefit was short‐lived and significant re‐accumulation of fluid was shown on his radiograph two weeks later (C). Further treatment with escalated doses of tPA/DNase failed to produce a response (D).

**Figure 2:**
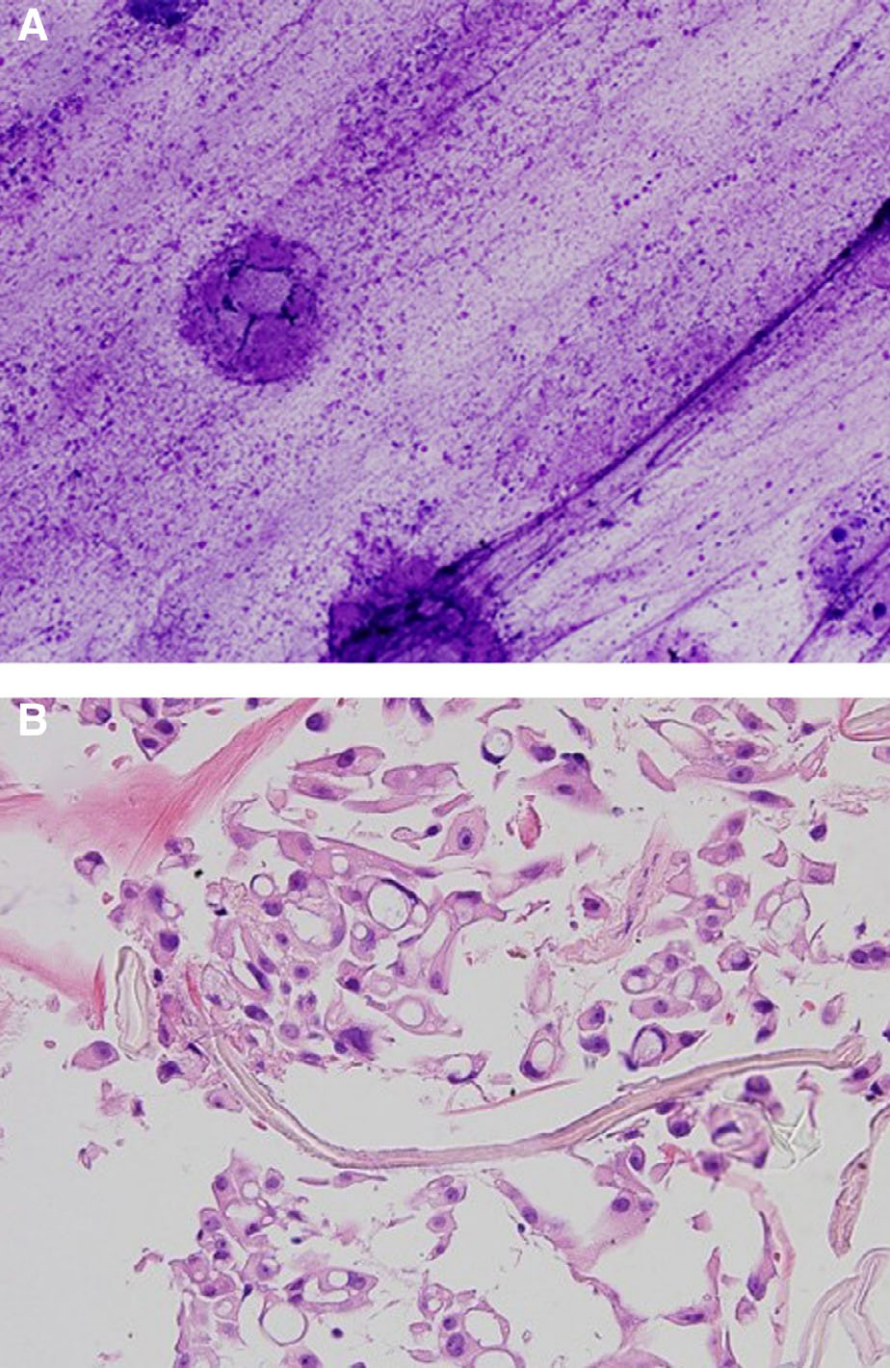
The cytology sample from Patient 1 demonstrated material consistent with hyaluronic acid (HA) both in intracellular and extracellular locations. The smear of the viscous pleural fluid (A) shows prominent extracellular granular material within the background consistent with HA (Diff‐Quick stain, original magnification, 10×). The cell block (B) of the same pleural fluid demonstrates malignant mesothelioma cells with prominent intracytoplasmic vacuoles containing eosinophilic material either with a globular appearance or an appearance similar to that of crinkled paper, both of which suggest the presence of hyaluronan (haematoxylin and eosin, original magnification, 40×).

Unfortunately, the fluid recurred over the next four weeks, resulting in a large multiloculated effusion associated with breathlessness and fatigue (Fig. 1A), necessitating hospitalization. Drainage via the IPC remained difficult because of the fluid viscosity. Four doses of intrapleural tissue plasminogen activator (tPA) 2.5 mg and DNase 5 mg were administered twice daily with good effect. A fluid sample was collected after the first dose (Sample 1) for viscosity and cytological analyses (Tables 1, 2 and Fig. 2, respectively). A total of 2.8 L of fluid was drained with significant radiographic improvement (Fig. 1B). However, the benefit was short‐lived and fluid re‐accumulated within two weeks (Fig. 1C), during which time he received one cycle of treatment in an immunotherapy trial. A further attempt with tPA 2.5 mg/DNase 5 mg given in outpatient clinic produced only marginal benefit. With strong manual suction via the IPC, 380 mL of viscous fluid was aspirated and a further 350 mL was drained the next day. Nonetheless, a massive residual effusion remained.

**Table 1:** Pleural fluid HA concentrations and viscosity levels (mPa·s) of pleural fluid after incubation with hyaluronidase (U/mL).

	HA concentration in pleural fluid	Fluid viscosity (mPa·s) after treatment with different concentrations of hyaluronidase (U/mL)			
		0	1	10	100
Patient 1 (Sample 1)	2.66×10^6^ ng/mL	800.21	54.62	21.91	2.94
Patient 1 (Sample 2)	5.39×10^6^ ng/mL	Too viscous	Too viscous	153.67	18.61
Patient 2 (Sample 3)	2.31×10^6^ ng/mL	303.03	168.01	6.59	2.87

The two samples from Patient 1 were taken about 38 days apart.

HA, hyaluronic acid.

**Table 2:** Pleural fluid viscosity levels (mPa·s) of pleural fluid after incubation with rhDNase.

	rhDNase (μg/mL)			
	0	2.5	5	10
Patient 1 (Sample 1)	780.56	690.93	627.61	567.38

Sample 1 was incubated with rhDNase at 2.5, 5, and 10 μg/mL at room temperature for 4 h before viscosity was measured. The range of concentration selected was to approximate the concentration of enzyme in a patient with a 1.5‐L pleural effusion treated with 5 mg of rhDNase.

rhDNase, recombinant human deoxyribonuclease.

The patient was readmitted two weeks later for ongoing difficulty with IPC drainage (<100 mL each attempt) (Fig. 1D). Sample 2 was collected for viscosity analyses (Table 1). During the hospitalization, he received intrapleural tPA 5 mg/DNase 5 mg and suction via the IPC with little effect (60 and 275 mL after the first two doses). A further attempt using tPA 10 mg and DNase 10 mg yielded 200 mL and minimal radiographic improvement. He was discharged and died seven months later from his mesothelioma.

### Patient 2

A 79‐year‐old female presenting with a right pleural effusion underwent video‐assisted thoracoscopic pleural biopsy (which showed mesothelioma) and talc poudrage. The fluid recurred after surgery necessitating a therapeutic aspirate which revealed extremely thick exudative pleural fluid. She underwent regular therapeutic aspiration in an outside hospital for her MPE over the next four years (during which time she received various systemic therapy) before she was referred to our centre for IPC insertion. The fluid was described as thick and “jelly‐like” and failed to drain via the IPC. Manual suction via the IPC (Rocket) was required for complete evacuation of the fluid. Talc slurry was administered after lung re‐expansion but the pleurodesis failed.

For the following 12 months, drainage was performed (roughly once weekly) via IPC. Sample 3 showed a typical specimen. Standard IPC suction bottles were ineffective to clear the thick fluid. Hence, the patient's carers were taught to perform IPC drainage using manual suction.

After one year, IPC drainage failed despite the above‐mentioned measures causing significant breathlessness necessitating a hospital admission. A trial of intrapleural tPA 2.5 mg/DNase 5 mg was successful and drained 1.5 L with significant radiographic clearance. Regular IPC drainage was re‐established. Her mesothelioma continued to progress and she died 10 months later.

### Fluid Viscosity and HA Analyses

Pleural fluid viscosity was determined as described by Simpson et al. [1]. HA concentrations were determined in the MPE supernatant using a commercially available HA binding assay (Corgenix, USA). To test the contribution of HA to the viscosity of the effusion, samples were incubated at room temperature for 10 min with hyaluronidase type V (0–100 U/mL) diluted in phosphate‐buffered saline (both Sigma‐Aldrich, USA). To model the effects of recombinant human DNase (rhDNase) treatment, the original pleural effusion sample was incubated in the laboratory with rhDNase (Pulmozyme®; Roche, USA) diluted in normal saline (Baxter, Australia) for 4 h before viscosity measurement.

We found that MPE from both patients had very high pleural fluid HA concentrations. Hyaluronidase, but not rhDNase, was effective in reducing the viscosity of the fluid in a dose‐dependent manner (Tables 1, 2, Video S1).

## Discussion

We described two mesothelioma patients with HA‐rich and extremely viscous MPE, limiting fluid drainage. Our laboratory work confirmed that HA, present in high concentrations in these fluids, likely contributed to the high viscosity which could be significantly reduced using hyaluronidase.

MPE from mesothelioma contains a vast number of important mediators. Mesothelioma has been known to express HA for over 80 years and HA pleural fluid concentrations are a valuable ancillary test for diagnosis. In our previous study including 96 mesothelioma patients, the pleural fluid HA level has an area under the curve of 0.89 in its diagnostic value of mesothelioma, highly comparable with mesothelin. Pleural fluid HA has also prognostic values [2]. Our experiments provide proof to the long suspicion that HA is involved in generating high viscosity in MPE.

HA is a long polymer of disaccharides up to 20,000 kDa in size produced by hyaluronan synthases, present in abundance in extracellular matrix. It can promote inflammation and angiogenesis—processes involved in MPE formation. HA is approved as intra‐articular treatment for osteoarthritis and for use in ophthalmologic conditions. It is also a common ingredient in skin care products [3].

HA can be degraded by hyaluronidases, a family of enzymes, and by various non‐enzymatic reactions (e.g. acidic/alkaline hydrolysis) [4]. Hyaluronidases have been used in low doses in various conditions, mainly to breakdown extracellular matrix and enhance drug distribution [5, 6]. Whether it can be safely applied intrapleurally and in sufficient therapeutic quantity to lyse HA in human MPE will need to be investigated.

At present, clinically available options for viscous MPE are limited. Use of tPA/DNase enhanced the evacuation of thick infected pleural fluid in clinical trials. The regime provided some short‐term benefits in Patient 1, but subsequent administration did not prove effective. Our ex vivo experiments did not find a major role of rhDNase in reducing the viscosity. As tPA is known to stimulate significant production of pleural fluid in healthy and diseased pleura, it is possible that the dilutional effect of this fluid formation helped with initial drainages. Whether tPA itself affects viscosity of MPE is unknown.

The composition of pleural fluid may be important to provide an informed clinical decision to ascertain an effective therapy for pleural effusion management. In cases of viscous fluid and high HA levels, strategies to counteract HA production or enhance its breakdown warrant exploration.

### Disclosure Statement

Appropriate written informed consent was obtained for publication of this case report and accompanying images.

## Supporting information


**Video S1.** Highly viscous pleural fluid from Patient 1 (Sample 2) in the tube labelled *untreated* is shown. Treatment of the same sample with hyaluronidase (100 U/mL for 10 min) significantly reduced the fluid viscosity as shown in the tube labelled *treated*.
